# Underuse of bystander defibrillation on females during out-of-hospital cardiac arrest: a retrospective observational study in Aotearoa New Zealand

**DOI:** 10.1016/j.resplu.2026.101252

**Published:** 2026-02-03

**Authors:** Verity F. Todd, Heather Hutchinson, Vinuli Withanarachchie, Andy Swain, Sarah Maessen, Aroha Brett, Bridget Dicker

**Affiliations:** a Paramedicine Research Unit, Paramedicine Department, School of Acute and Primary Health, Faculty of Health and Environmental Sciences, Auckland University of Technology, 640 Great South Road, Manukau City Centre, Auckland 2025, New Zealand; b Clinical Evaluation, Research, and Insights Team, Hato Hone St John, Building A Level 3/600 Great South Road, Ellerslie, Auckland 1051, New Zealand; c Wellington Free Ambulance Service, 19 Davis Street, Pipitea, Wellington 6011, New Zealand; d Māori Health and Equity Team, Kahui Mauaka, Building A Level/600 Great South Road, Ellerslie, Auckland 1051, New Zealand

**Keywords:** Out-of-hospital cardiac arrest, Bystander defibrillation, AED, Prehospital, Equity, Women, Bystander CPR

## Abstract

**Introduction:**

Early bystander CPR and defibrillation improve cardiac arrest survival. International studies show women receive fewer bystander interventions after out-of-hospital cardiac arrest (OHCA) than men. We investigated associations between sex and bystander interventions in OHCA in Aotearoa New Zealand.

**Methods:**

Retrospective cohort study of adult OHCA with attempted resuscitation (January 2019–December 2023). Variables included demographics, ethnicity, socioeconomic deprivation, rurality, location type, and witness status. Logistic regression assessed associations between sex and bystander CPR and bystander defibrillation (shock delivered), adjusting for covariates.

**Results:**

Of 9377 OHCA events, 29.6% occurred in females. Bystander CPR rates were similar between females (75.7%) and males (77.3%) (AOR 1.06, 95% CI: 0.94–1.18, *p* = 0.36). However, bystander defibrillation occurred in only 1.9% of females versus 5.4% of males. Females had 65% lower odds of receiving bystander defibrillation in unadjusted analysis (UOR 0.35, 95% CI: 0.26–0.47, *p* < 0.001) and 39% lower odds after adjustment (AOR 0.61, 95% CI: 0.44–0.84, *p* = 0.002). Among shockable rhythm cases, sex disparity was not significant (AOR 0.83, 95% CI: 0.59–1.15, *p* = 0.26).

**Conclusion:**

Despite equitable bystander CPR rates, females had significantly lower odds of receiving bystander defibrillation. In shockable rhythm cases, the disparity was not significant after adjustment for covariates, suggesting that patient and event characteristics mediate rather than confound this relationship. Findings highlight the need for targeted interventions addressing strategies to improve bystander defibrillation for females in cardiac arrest.

## Highlights

• High overall bystander CPR rates (76.9%) with no sex-based differences.
• Only 1.9% of females received defibrillation vs 5.4% of males in cardiac arrest.
• Females had 39% lower odds of bystander defibrillation despite equal CPR rates.
• In shockable rhythms, patient and event characteristics mediate the sex disparity in bystander defibrillation.
• Ethnicity and deprivation create layered inequities in emergency cardiac care.

## Introduction

Out-of-hospital cardiac arrest (OHCA) is a time-critical medical emergency where survival is closely tied to immediate intervention. Survival following OHCA is contingent on the ‘chain of survival’, particularly its three early links: recognition of cardiac arrest and alerting emergency medical services (EMS), high-quality cardiopulmonary resuscitation (CPR), and rapid defibrillation.^1^

It is well established that early bystander CPR and defibrillation with an automated external defibrillator (AED) improve the odds of surviving a cardiac arrest.^2^, ^3^ The rates of bystander interventions vary internationally but are increasing.^4^, ^5^, ^6^ There exists a significant sex disparity in bystander defibrillation, with women often receiving fewer public interventions after OHCA compared to men.^7^, ^8^, ^9^, ^10^, ^11^ While evidence is equivocal when it comes to CPR initiation, a recent meta-analysis of almost 500,000 OHCA demonstrated that women had 21% lower odds of receiving bystander AED application during a cardiac arrest compared to men.^9^ In addition, women experiencing a public OHCA in racially diverse neighbourhoods were significantly less likely to receive bystander defibrillation, i.e. 12% less likely in Black neighbourhoods and 26% less likely in predominantly Hispanic areas.^12^ These findings highlight layered inequities in emergency responses faced by women experiencing OHCA from racial and ethnic minority groups.

These healthcare equity challenges also exist in Aotearoa New Zealand (AoNZ), disproportionately affecting Māori (the indigenous people of AoNZ) and Pacific peoples.^13^, ^14^ Higher rates of OHCA are reported for Māori and Pacific peoples compared to those of European or other ethnicities (144 and 114 versus 94 per 100,000 incidents per year, respectively), with lower odds of survival in both Māori and Pacific peoples (39% and 48%).^15^ Research examining sex-based disparities in bystander interventions within the unique demographic and geographic context of Aotearoa New Zealand remains limited.

This study aimed to investigate whether patient sex is associated with receipt of bystander CPR and bystander defibrillation in out-of-hospital cardiac arrest in AoNZ, using five years of national prehospital OHCA registry data from a setting with universal healthcare coverage and distinct demographic characteristics.

## Methods

### Study design

We undertook a retrospective cohort study investigating the association between bystander CPR or defibrillation and patient sex (male or female) in out-of-hospital cardiac arrest. A bystander is defined as a person who is on scene or alerted but not dispatched as part of an organised emergency response system, and includes alerted volunteer responders (GoodSAM responders,^16^ off-duty medical personnel, and Police.^17^ Bystander CPR is defined according to Utstein criteria.^17^ In this study, bystander defibrillation is defined as a shock(s) delivered by a public access or privately owned defibrillator by a bystander (as defined above). This definition of bystander defibrillation is distinct from the Utstein variable of ‘Bystander AED use’,^17^ defined as an AED applied by a bystander, regardless of whether a shock was delivered. Bystander AED use is not currently recorded in the AoNZ OHCA Registry.

### Setting

AoNZ has a population of approximately 5.1 million. Nationally, two road-based EMS providers (Hato Hone St John (HHStJ) and Wellington Free Ambulance (WFA)) provide emergency ambulance care. HHStJ provides EMS to 90% of the AoNZ population, with the remainder serviced by WFA. Regional helicopter-based emergency medical services are not included in this study.

In AoNZ, Fire and Emergency New Zealand (FENZ) are dispatched to OHCA events alongside EMS as first responders equipped to deliver CPR and defibrillation with an automatic external defibrillator. On-duty responders dispatched as part of an organised emergency response system, including FENZ, were not included as bystanders in this study.

### Inclusion and exclusion criteria

ANZPaCC captures all clinical information from incidents attended by road-based EMS in AoNZ and is linked to Manatū Hauora (Ministry of Health) data using a patient-specific National Health Index (NHI).^18^, ^19^

**Fig. 1:**
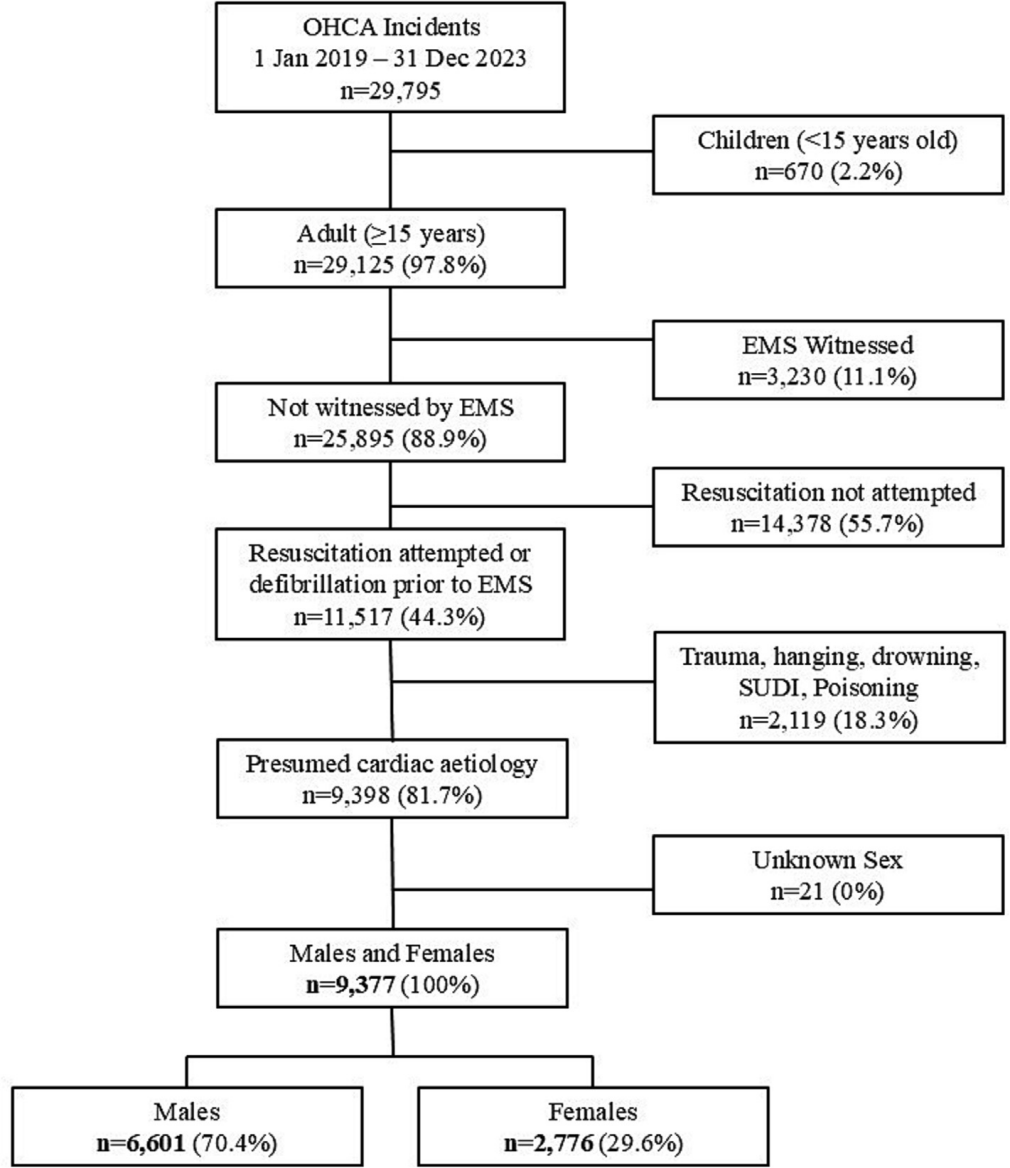
**Cohort flowchart of out-of-hospital cardiac arrest (OHCA) cases in males and females meeting the inclusion criteria**. EMS – Emergency Medical Services, SUDI – sudden unexpected death in infancy.

All ANZPaCC incidents between 1 January 2019 and 31 December 2023 were included. All adult (≥15 years old, as defined by AoNZ health service classifications) OHCA patients who received attempted resuscitation by EMS or defibrillation prior to ambulance arrival were included. Arrests with a non-cardiac aetiology and those witnessed by EMS personnel were excluded, as were those where the patient’s sex was unknown (Fig. 1).

### Variables

The data variables for ANZPaCC are described in the ambulance care standard and the Manatū Hauora data dictionaries.^18^, ^19^ ANZPaCC data is derived from structured electronic patient report forms with mandatory field completion for all OHCA cases. Core Utstein variables, including witnessed status, bystander CPR, and bystander defibrillation, must be completed before case closure, ensuring complete data capture for these variables. Witnessed status is categorised as 'bystander witnessed', 'EMS witnessed', or 'unwitnessed' based on paramedic scene assessment and bystander interviews; cases where witnessed status could not be definitively determined are coded as 'unwitnessed'. Bystander interventions (CPR and defibrillation) are documented based on bystander reports and scene findings.

Ethnicity data were obtained through linkage of ANZPaCC records to Manatū Hauora (Ministry of Health) national health data via each patient's unique NHI number. Ethnicity is self-identified by individuals during routine healthcare encounters and recorded in national health records. Prioritised ethnicity was assigned for each case following the Aotearoa New Zealand Ethnicity Data Protocols (HISO 10001:2017), which uses a hierarchy (Māori > Pacific Peoples > Asian > Middle Eastern/Latin American/African > Other > European) to ensure visibility of Māori and Pacific peoples in health data when individuals identify with multiple ethnic groups.^20^ The NHI linkage rate for OHCA cases in ANZPaCC is consistently > 95%, ensuring high-quality ethnicity data capture. Cases without ethnicity data (*n* = 473, 5.0%) represent instances where NHI linkage was unsuccessful or ethnicity was not recorded in national health records. The ‘European and Other’ category contains cases from all ethnicity groups except Māori, Pacific peoples, or Residual (unknown/unstated).

Rurality based on the incident location was grouped using the Geographical Classification for Health which reflects access to healthcare, whereby U1 and U2 were grouped as ‘Urban’ and R1-R3 as ‘Rural’.^21^

Socioeconomic deprivation was measured using the New Zealand Index of Deprivation 2018 (NZDep2018) linked to each patient's residential address, with decile scores grouped into quintiles where Quintile 1 represents the least deprived areas and Quintile 5 the most deprived areas.^22^ Socioeconomic deprivation was measured using the New Zealand Deprivation Index 2018 (NZDep2018) based on case home address rather than event location. This approach was chosen because home address deprivation reflects chronic socioeconomic circumstances that influence overall health status and cardiac arrest risk. The deprivation quintile (NZDep2018) was included in multivariable models as a categorical variable, with the least deprived quintile (Q1) as the reference category. Location type was further grouped as Home, Public (including road and footpath), and Other (including Aged Care and Healthcare Facilities). Minutes to First Arrival was the time from the emergency call pick-up to the arrival of the first dispatched EMS or FENZ vehicle on scene. First arresting rhythm was categorised as shockable or non-shockable based on the first assessment of a cardiac rhythm by a defibrillator or cardiac monitor.

### Statistical analysis

Patient and incident characteristics were compared between male and female patients using the chi-square test for categorical variables and the Mann-Whitney *U* test for continuous variables. For categorical variables with significant chi-square results, *z*-tests for column proportions were used post-hoc to identify specific categories that differed significantly between males and females. Extreme values were excluded from the analysis (age ≥105 years, minutes to arrival ≥100 min). A pairwise deletion approach was used. Multivariable binary logistic regression models examined associations between patient and event characteristics and bystander interventions. Firth's penalised likelihood logistic regression was used for adjusted bystander CPR and defibrillation analyses to address potential bias from rare events and separation issues. This approach provides less biased estimates when outcome prevalence is low. Sex was the dependent variable, with models adjusted for age, ethnicity, witnessed status, event location type, rurality, and socioeconomic deprivation via forced entry. These factors were included in the adjusted model based on existing evidence for relationships with bystander interventions. An additional analysis restricted to cases with a first arresting shockable rhythm was undertaken using the same statistical approach.

The variables included in our adjusted models (age, ethnicity, witnessed status, event location type, rurality, and socioeconomic deprivation) were selected based on existing literature demonstrating associations with bystander interventions. We acknowledge that several of these variables, particularly event location and witness status, likely function as mediators rather than confounders in the relationship between sex and bystander defibrillation. The adjusted analyses describe changes in effect estimates after accounting for these potential mediating pathways rather than isolating a causal effect of sex. The unadjusted odds ratios represent the total real-world disparity, while adjusted odds ratios reflect residual direct effects after accounting for mediating variables.

Results are presented as unadjusted and adjusted odds ratios (ORs) with 95% confidence intervals (CIs). Analyses were conducted using SPSS version 30.0 and SAS 9.4 (Firth penalised logistic regression), and a *p*-value of <0.05 was considered statistically significant.

## Results

During this five-year period, 9377 OHCA events met the inclusion criteria (adult, not EMS-witnessed, resus attempted, presumed cardiac cause) (Fig. 1). Overall, 29.6% (*n* = 2776) of included OHCA events occurred in females, whilst 70.4% (*n* = 6601) occurred in males (Table 1).

**Table 1:** Descriptive statistics of all presumed cardiac OHCA events (excluding EMS-witnessed and resuscitation not attempted cases).

		**Total**	**Female**	**Male**	***P*-value**
OHCA Events		9377	2776 (29.6%)	6601 (70.4%)	
Age Median [IQR]		67 [56–77]	69 [56–79]	67 [56–77]	< 0.001
Ethnicity	European and Other	6022 (67.6%)	1657 (62.6%)	4365 (69.8%)	< 0.001
	Māori	2017 (22.7%)	692 (26.1%)	1325 (21.2%)	
	Pacific Peoples	865 (9.7%)	300 (11.3%)	565 (9.0%)	
	*Missing*	473 (5.0%)			
Rurality	Urban	7084 (76.5%)	2107 (76.7%)	4977 (76.5%)	0.837
	Rural	2172 (23.5%)	641 (23.3%)	1531 (23.5%)	
	*Missing*	121 (1.3%)			
Deprivation (Patient address)	Q1 (least deprived)	1246 (13.7%)	301 (11.1%)	945 (14.8%)	< 0.001
	Q2	1408 (15.5%)	370 (13.6%)	1038 (16.2%)	
	Q3	1621 (17.8%)	456 (16.8%)	1165 (18.2%)	
	Q4	1913 (21.0%)	586 (21.6%)	1327 (20.8%)	
	Q5 (most deprived)	2923 (32.1%)	1003 (36.9%)	1920 (30.0%)	
	*Missing*	266 (2.8%)			
Location type	Home	6828 (74.5%)	2244 (82.5%)	4584 (71.2%)	< 0.001
	Public	1926 (21.0%)	299 (11.0%)	1627 (25.3%)	
	Other (Including Aged Care and Healthcare Facilities)	406 (4.4%)	176 (6.5%)	230 (3.6%)	
	*Missing*	217 (2.3%)			
Minutes to first arrival median [IQR]		8.6 [6.6–11.6]	8.4 [6.6–11.4]	8.6 [6.7–11.7]	0.036
	*Missing*	91 (1.0%)			
First arresting rhythm	Non-shockable	5126 (54.7%)	1870 (67.4%)	3256 (49.3%)	< 0.001
	Shockable	4096 (43.7%)	844 (30.4%)	3252 (49.3%)	
	Unknown	155 (1.7%)	62 (2.2%)	93 (1.4%)	
Bystander witnessed	No	3816 (40.7%)	1245 (44.8%)	2571 (38.9%)	< 0.001
	Yes	5561 (59.3%)	1531 (55.2%)	4030 (61.1%)	
Bystander CPR	No	2171 (23.1%)	674 (24.3%)	1497 (22.7%)	0.093
	Yes	7206 (76.9%)	2102 (75.7%)	5104 (77.3%)	
Bystander defibrillation	No	8969 (95.6%)	2722 (98.1%)	6247 (94.6%)	< 0.001
	Yes	408 (4.4%)	54 (1.9%)	354 (5.4%)	

*P*-values from chi-square tests for categorical variables and Mann-Whitney *U* test for continuous variables with a significance threshold of *p* < 0.05. OHCA – out-of-hospital cardiac arrest; IQR – interquartile range; Q1–Q5 – Quintile 1 (least deprived) to Quintile 5 (most deprived); CPR – cardiopulmonary resuscitation.

### Descriptive analysis of OHCA cases stratified by sex

Compared to males, females suffering OHCA were slightly older, a higher proportion identified as ethnic minority groups, and more resided in areas of high deprivation (*p* < 0.001; Table 1). A lower proportion of females arrested in public locations, had an OHCA witnessed by a bystander, and presented with a shockable rhythm (*p* < 0.001; Table 1). A descriptive table of the OHCA cases with a shockable first arresting rhythm is presented in Supplementary Table 1.

### Sex differences in bystander CPR

**Fig. 2:**
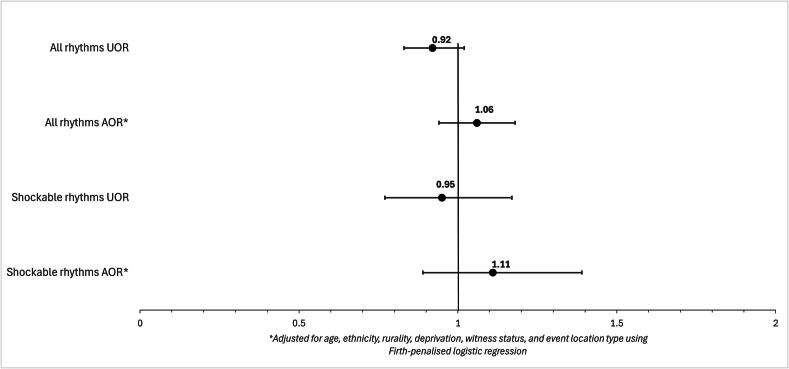
**Odds ratios for females compared to males for receiving bystander CPR. Adjusted and unadjusted odds ratios are presented for the all arresting rhythms group and the shockable first arrest rhythm subgroup, with males as the reference group. The Firth penalised logistic regression model adjusted for age, ethnicity, rurality, deprivation, witness status, and event location type. Values <1.0 indicate reduced odds for females; values >1.0 indicate increased odds for females. Error bars are 95% confidence intervals**. UOR – unadjusted odds ratio; AOR – adjusted odds ratio.

Bystander CPR rates were similar between females (75.7%, *n* = 2102) and males (77.3%, *n* = 5104; *p* = 0.093) (Table 1). Patient sex was not significantly associated with receipt of bystander CPR in unadjusted analysis of first arresting rhythm cases (OR 0.92, 95% CI: 0.83–1.02, *p* = 0.097), and this remained non-significant after adjusting for age, ethnicity, rurality, deprivation, witness status, and event location type (AOR 1.06, 95% CI: 0.94–1.18, *p* = 0.36) (Fig. 2). Similarly, in the subgroup with shockable first arresting rhythms, there was no significant difference in bystander CPR between females and males in either unadjusted (OR 0.95, 95% CI: 0.77–1.17, *p* = 0.600) or adjusted analyses (AOR 1.11, 95% CI: 0.89–1.39, *p* = 0.360) (Fig. 2, Supplementary Tables 2 and 3). Odds ratios for additional associations with bystander CPR for demographic and event characteristics are available in Supplementary Table 2 (all arresting rhythms) and Supplementary Table 3 (shockable first arresting rhythms).

### Sex differences in bystander defibrillation

**Fig. 3:**
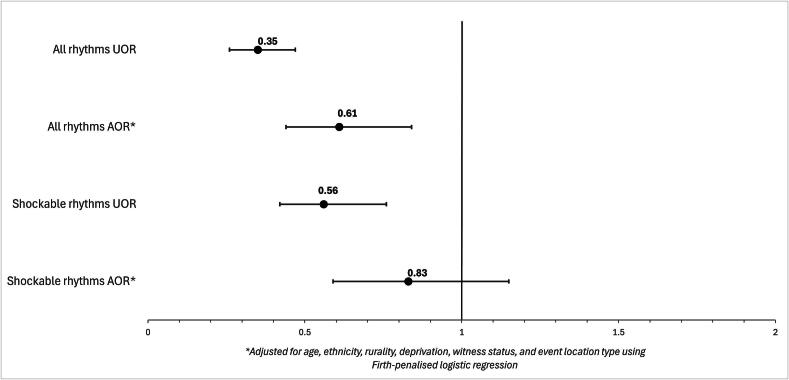
**Odds ratios for females compared to males for receiving bystander defibrillation. Adjusted and unadjusted odds ratios are presented for the all arresting rhythms group and the shockable first arrest rhythm subgroup, with males as the reference group. The Firthpenalised logistic regression model adjusted for age, ethnicity, rurality, deprivation, witness status, and event location type. Values <1.0 indicate reduced odds for females; values >1.0 indicate increased odds for females. Error bars are 95% confidence intervals**. UOR – unadjusted odds ratio; AOR – adjusted odds ratio.

The overall rate of bystander defibrillation was 4.4% (*n* = 408). Bystander defibrillation was significantly higher in males (5.4%, *n* = 354) than females (1.9%, *n* = 54) (*p* < 0.001; Table 1). Females presenting in any arresting rhythm were 65% less likely to receive bystander defibrillation in the unadjusted model (UOR 0.35, 95% CI: 0.26–0.47, *p* < 0.001) and 39% less likely to receive bystander defibrillation than males in the adjusted model (AOR 0.61, 95% CI: 0.44–0.84, *p* = 0.002) (Fig. 3, Supplementary Table 4). Among cases with shockable first arresting rhythms, females remained less likely to receive bystander defibrillation in unadjusted analysis (UOR 0.56, 95% CI: 0.42–0.76, *p* < 0.001) (Fig. 3; Supplementary Table 5). However, after adjusting for age, ethnicity, rurality, deprivation, arrest location and witness status, this association was not significant (AOR 0.83, 95% CI: 0.59–1.15, *p* = 0.26) (Fig. 3, Supplementary Table 5).

### Factors associated with bystander interventions

Associations between other OHCA case characteristics and bystander CPR in all arresting rhythms are shown in Supplementary Table 2. Higher odds of receiving bystander CPR were associated with younger age, Māori ethnicity, rural location, occurring in a public location, and being witnessed by a bystander (*p* < 0.05). Lower odds of receiving bystander CPR were associated with residing in the most deprived areas (NZDep Quintile 5). Similar associations with bystander CPR were observed in cases with a shockable first arresting rhythm (Supplementary Table 3).

Associations between other OHCA case characteristics and bystander defibrillation in all arresting rhythms are shown in Supplementary Table 4. Higher odds of receiving bystander defibrillation were associated with rural locations, public locations, and being witnessed by a bystander (*p* < 0.05). Lower odds of receiving bystander defibrillation were associated with female sex, older age, and residing in the most deprived areas (NZDep Quintile 5) (*p* < 0.05). There was no significant association between ethnicity and receiving bystander defibrillation (*p* = 0.45). Associations between other OHCA case characteristics with bystander defibrillation in cases with first arresting shockable rhythms are shown in Supplementary Table 5.

## Discussion

Despite similar rates of bystander CPR between sexes, there is a significant sex-based disparity in bystander defibrillation during OHCA in AoNZ, with females 39% less likely to receive this life-saving intervention. However, in cases presenting with a first arresting shockable rhythm, adjusting for variables including age, ethnicity, rurality, deprivation, witness status, and location type substantially reduced the sex disparity. This suggests that these covariates, particularly arrest location and witness status, function as mediators rather than confounders, explaining the mechanism underlying disparity. The unadjusted odds ratio (UOR 0.35, 95% CI: 0.26–0.47, *p* < 0.001; for defibrillation across all rhythms) reflects the total real-world sex disparity, while the adjusted odds ratio (AOR 0.61, 95% CI: 0.44–0.84, *p* = 0.002) reflects residual direct effects after accounting for these mediating pathways. The defibrillation disparity (1.9% vs 5.4%) represents a critical equity gap that compounds existing disadvantages faced by women experiencing cardiac arrest in AoNZ. In examining other factors associated with bystander interventions, our study also demonstrated that higher odds of bystander CPR and defibrillation were associated with OHCA occurring in a public location, with lower odds associated with residing in the most socioeconomically deprived areas.

In general, women have poorer odds of surviving a cardiac arrest.^23^, ^24^ The survival disadvantage appears to stem from multiple compounding factors: women present with less favourable arrest characteristics (older age, fewer shockable rhythms, less witnessed arrests), receive fewer life-saving interventions during the acute event (including bystander interventions and access to prehospital critical care,^25^ and experience disparities in post-resuscitation care, including reduced access to coronary angiography and targeted temperature management.^23^, ^26^ These reflect cumulative discrepancies between males and females in the cardiac arrest chain of survival.

We observed no sex-based difference in bystander CPR rates, adding to the equivocal international evidence.^9^ The high overall bystander CPR rate in Aotearoa New Zealand (76.9%) and the equity in CPR provision across sex groups may reflect the effectiveness of national CPR training initiatives and dispatcher-assisted CPR protocols.

The sex disparity in bystander defibrillation in AoNZ aligns with a recent meta-analysis reporting that women in OHCA had 21% lower odds of a bystander applying an AED.^9^ The gender gap in bystander defibrillation is widening.^10^ Despite a clear pattern of lower bystander defibrillation rates for women internationally, there is significant heterogeneity between studies, indicating that explanations for the effect may include geographic-, system-, or culture-specific aspects.^7^, ^8^, ^9^, ^27^, ^28^, ^29^

Our analysis included all first arresting rhythms rather than restricting to shockable rhythms. The factors influencing the decision to apply an AED, including patient characteristics, witness status, and arrest location, operate upstream of rhythm determination.^6^, ^30^ Restricting analysis to shockable rhythms introduces selection bias, as females are less likely to present with shockable rhythms,^10^, ^23^, ^31^, ^32^ which is a significant contributor to the survival discrepancy between sexes.^32^ These rhythm differences persist even in the cases with the most favourable resuscitation conditions (public location, witnessed, bystander CPR), suggesting the contribution of underlying biological differences between men and women.^31^ Impacting on shockable rhythm is the underlying aetiology, including a lower prevalence of underlying coronary artery disease in women.^31^

Location was strongly associated with bystander defibrillation, with individuals who arrested in public locations over 10 times more likely to receive defibrillation than those who arrested at home (AOR 10.51, 95% CI: 8.13–13.58). This disparity reflects the strategic placement of AEDs in public spaces, in contrast to the limited availability of defibrillators in residential settings.^33^, ^34^, ^35^ The location effect mediates existing sex disparities, given that 82.5% of female arrests occurred at home versus 71.2% of male arrests, consistent with international studies.^23^ This effectively creates a layered disadvantage, where women are both less likely to arrest in AED-accessible locations and less likely to receive bystander defibrillation when it occurs. These findings underscore the urgent need for innovative AED accessibility strategies, including smartphone-activated volunteer responder systems that can rapidly deliver AEDs to homes.^16^, ^36^, ^37^, ^38^

Deprivation has been associated with poorer OHCA outcomes internationally.^32^, ^39^, ^40^ This association is compounded by inequitable AED distribution, with public access defibrillators demonstrably less accessible in areas of higher deprivation.^41^, ^42^, ^43^, ^44^, ^45^ We reveal significant associations between socioeconomic deprivation and bystander interventions, with individuals from the most deprived areas experiencing 50% lower odds of receiving both bystander CPR and bystander defibrillation compared to those from the least deprived areas, consistent with other studies.^46^, ^47^ The association between deprivation and bystander defibrillation likely reflects both individual socioeconomic factors (health literacy, cultural and social considerations, community engagement) associated with home address and resourcing (AED availability, community resources) associated with event location.^6^, ^30^ We have previously shown that Māori and Pacific peoples experience cardiac arrest more frequently and have poorer survival outcomes.^15^ These deprivation‑related inequities are particularly concerning given that Māori and Pacific communities in AoNZ disproportionately experience socioeconomic disadvantage. This intersection of sex, ethnicity, and deprivation creates layered disadvantages, with women from ethnic minority groups facing multiple compounding barriers to receiving bystander defibrillation.^12^

### Limitations

Several limitations should be acknowledged in this study. Our analysis focused on defibrillation delivery rather than AED pad placement or attempted AED use (i.e. the Utstein variable ‘bystander AED use’,^17^ which may underestimate cases where bystanders retrieved and prepared an AED but did not deliver a shock due to non-shockable rhythms or other factors. While the mandatory data entry system ensures complete capture of bystander intervention variables, some misclassification may occur in cases where paramedics could not definitively determine intervention status from scene information. This would likely bias our results toward the null, suggesting our observed disparities may be conservative estimates.

Our adjusted analyses should be interpreted with caution when making causal inferences. Several covariates included in our models, particularly event location and witness status, likely function as mediators on the causal pathway between sex and bystander interventions rather than true confounders. Future mediation analysis would be required to fully quantify the extent to which location, witness status, and other factors mediate the sex-defibrillation relationship.

There was information about bystander characteristics, including the number of bystanders present, training background, relationship to the patient, and specific barriers encountered, which limits our ability to identify targeted intervention strategies. We were unable to distinguish between bystander-initiated CPR and call-centre-initiated CPR.

The data presented in this study encompassed the COVID-19 period. Although bystander CPR and OHCA survival were not significantly reduced during the COVID-19 period in AoNZ, other bystander interventions may have been impacted.^48^

Only cases where EMS initiated a resuscitation (or where defibrillation occurred prior to EMS arrival) were included in this study. If there are biases in resuscitation decision-making, e.g. by ethnicity, deprivation, sex, or location, this may have introduced selection bias in our cohort.

Deprivation scores were applied based on the patient’s home address. While home address deprivation captures chronic socioeconomic disadvantage associated with cardiac arrest risk and comorbidity, event location deprivation may more directly reflect local emergency response infrastructure, including public AED placement and density. However, since 74.5% of arrests occurred at home, these measures are applicable in most cases. For the 25.5% of arrests that occur outside the home, home and event location deprivation may differ, potentially leading to misclassification.

Lastly, while we used prioritised ethnicity classifications, we acknowledge that ethnicity data may not fully capture the diversity within Māori and Pacific peoples populations.

## Conclusion

Despite high overall bystander CPR rates with no sex-based differences, disparity was found in defibrillation rates between females and males (1.9% vs 5.4%, respectively). This represents a critical equity gap that compounds existing disadvantages faced by women experiencing cardiac arrest in AoNZ. Among shockable rhythm cases, the defibrillation sex disparity was not statistically significant after adjustment, suggesting that patient and event characteristics likely mediate this relationship. These findings highlight the need for targeted strategies to improve recognition and response to female cardiac arrest, particularly in high-deprivation and ethnic minority populations.

## Funding source

Publication costs were supported by the Australasian College of Paramedicine Research Impact Grant (Verity Todd, 2025) and the AUT Faculty of Health and Environmental Sciences Rolling Fund (2026).

## Declaration of generative AI and AI-assisted technologies in the manuscript preparation process

During the preparation of this work, the lead author used Claude (Anthropic) to assist with literature searching and manuscript editing. After using this tool/service, the authors reviewed and edited the content as needed and take full responsibility for the content of the published article.

## CRediT authorship contribution statement

**Verity F. Todd:** Writing – review & editing, Writing – original draft, Visualization, Resources, Methodology, Investigation, Data curation, Conceptualization. **Heather Hutchinson:** Writing – review & editing, Writing – original draft, Visualization, Methodology, Investigation, Formal analysis, Data curation, Conceptualization. **Vinuli Withanarachchie:** Writing – review & editing, Writing – original draft, Visualization, Investigation. **Andy Swain:** Writing – review & editing, Writing – original draft, Methodology, Investigation, Conceptualization. **Sarah Maessen:** Writing – review & editing, Writing – original draft, Methodology, Investigation. **Aroha Brett:** Writing – review & editing, Visualization, Methodology, Investigation, Conceptualization. **Bridget Dicker:** Writing – review & editing, Writing – original draft, Visualization, Supervision, Resources, Project administration, Methodology, Investigation, Formal analysis, Data curation, Conceptualization.

## Ethics approval

Ethics approval was granted for research using the Aotearoa New Zealand Paramedic Care Collection (ANZPaCC) from the Northern B Health and Disability Ethics Committee (2022 FULL 13415). Locality approval was provided by Hato Hone St John (HHStJ) and Wellington Free Ambulance (WFA).

## Declaration of competing interest

HH, VW, SM, and AB are employees of HHStJ. AS is an employee of WFA.

## Glossary of abbreviations

AED: Automated External Defibrillator
ANZPaCC: Aotearoa New Zealand Paramedic Care Collection
AOR: Adjusted Odds Ratio
CI: Confidence Interval
CPR: Cardiopulmonary Resuscitation
EMS: Emergency Medical Services
FENZ: Fire and Emergency New Zealand
HHStJ: Hato Hone St John
IQR: Interquartile Range
NHI: National Health Index (patient identifier)
NZ: Aotearoa New Zealand
NZDep: New Zealand Index of Deprivation
NZDep2018: New Zealand Index of Deprivation 2018
OHCA: Out-of-Hospital Cardiac Arrest
OR: Odds Ratio
Q1–Q5: Quintile 1 to Quintile 5 (deprivation quintiles)
ROSC: Return of Spontaneous Circulation
SPSS: Statistical Package for the Social Sciences (statistical software)
STROBE: Strengthening the Reporting of Observational Studies in Epidemiology
UOR: Unadjusted odds ratio
WFA: Wellington Free Ambulance
