# Supplementary material for: Underuse of bystander defibrillation on females during out-of-hospital cardiac arrest: a retrospective observational study in Aotearoa New Zealand
Source: Resusc Plus. 2026 Feb 3;28:101252. doi: 10.1016/j.resplu.2026.101252 (PMC12924750; doi:10.1016/j.resplu.2026.101252)
Supplement: Supplementary Tables [file mmc1.docx]

##### Supplementary Table 1 – Descriptive statistics of presumed cardiac OHCA events (excluding EAS-witnessed and no resus attempted) presenting in a Shockable rhythm only

|  |  | Total | Female | Male | P-value |
| --- | --- | --- | --- | --- | --- |
| OHCA Events |  | 4,096 | 844 (20.6%) | 3252 (79.4%) |  |
| Age Median [IQR] |  | 65.0 [55.0-75.0] | 64.5 [53.0-76.0] | 65.0 [55.0-74.0] | .497 |
| Ethnicity | European and Other | 2,656 (68.2%) | 490 (60.3%) | 2,166 (70.3%) | <.001 |
|  | Māori | 869 (22.3%) | 233 (28.7%) | 636 (20.6%) |  |
|  | Pacific Peoples | 368 (9.5%) | 90 (11.1%) | 278 (9.0%) |  |
|  | *Missing* | 203 (5.0%) |  |  |  |
| Rurality | Urban | 3,104 (76.9%) | 635 (75.9%) | 2,469 (77.1%) | .439 |
|  | Rural | 934 (23.1%) | 202 (24.1%) | 732 (22.9%) |  |
|  | *Missing* | 58 (1.4%) |  |  |  |
| Deprivation (Patient address) | Q1 (least) | 632 (15.9%) | 103 (12.5%) | 529 (16.8%) | <.001 |
|  | Q2 | 655 (16.5%) | 104 (12.6%) | 551 (17.5%) |  |
|  | Q3 | 703 (17.7%) | 140 (17.0%) | 563 (17.9%) |  |
|  | Q4 | 821 (20.7%) | 178 (21.6%) | 643 (20.4%) |  |
|  | Q5 (most) | 1161 (29.2%) | 300 (36.4%) | 861 (27.4%) |  |
|  | *Missing* | 124 (3.0%) |  |  |  |
| Location Type | Home | 2,546 (64.1%) | 612 (74.5%) | 1,934 (61.4%) | <.001 |
|  | Public | 1,291 (32.5%) | 164 (20.0%) | 1,127 (35.8%) |  |
|  | Other (Including Aged Care and Healthcare Facilities) | 133 (3.4%) | 45 (5.5%) | 88 (2.8%) |  |
|  | Missing | 126 (3.1%) |  |  |  |
| Minutes to First Arrival Median [IQR] |  | 8.4 [6.5-11.4] | 8.2 [6.4-10.9] | 8.5 [6.6-11.5] | <.05 |
|  | *Missing* | 50 (1.2%) |  |  |  |
| Bystander Witnessed | No | 988 (24.1%) | 199 (23.6%) | 789 (24.3%) | .679 |
|  | Yes | 3,108 (75.9%) | 645 (76.4%) | 2,463 (75.7%) |  |
| Bystander CPR | No | 604 (14.7%) | 129(15.3%) | 475 (14.6%) | .621 |
|  | Yes | 3,492 (85.3%) | 715 (84.7%) | 2,777 (85.4%) |  |
| Bystander defibrillation | No | 3,688 (90.0%) | 790 (93.6%) | 2,898 (89.1%) | <.001 |
|  | Yes | 408 (10.0%) | 54 (6.4%) | 354 (10.9%) |  |

P-values from chi-square tests for categorical variables and Mann-Whitney U test for continuous variables with a significance threshold of p<.05. OHCA – out-of-hospital cardiac arrest; IQR – interquartile range; Q1-Q5 – Quintile 1 to Quintile 5; CPR – cardiopulmonary resuscitation.

##### Supplementary Table 2 - Associations with Bystander CPR in all first arresting rhythm cases

|  |  | Unadjusted Odds Ratio (uOR) (95%CI) | uOR P-value | Adjusted Odds Ratio^#^ (aOR) (95%CI) | aOR P-value |
| --- | --- | --- | --- | --- | --- |
| Sex | Female | 0.92 (0.83-1.02) | .097 | 1.06 (0.94-1.18) | .360 |
|  | Male (ref) | 1.00 |  | 1.00 |  |
| Age | (Years) | 0.98 (0.98-0.99) | <.001 | 0.98 (0.98-0.99) | <.001 |
| Ethnicity | European and Other (ref) | 1.00 | .270 | 1.00 | <.05 |
|  | Māori | 1.10 (0.98-1.25) |  | 1.17 (1.02-1.35) |  |
|  | Pacific Peoples | 1.05 (0.89-1.24) |  | 1.17 (0.97-1.42) |  |
| Rurality | Urban (ref) | 1.00 | <.05 | 1.00 | <.001 |
|  | Rural | 1.18 (1.05-1.33) |  | 1.27 (1.12-1.44) |  |
| Deprivation (Patient address) | Q1 (least) (ref) | 1.00 | <.001 | 1.00 | <.001 |
|  | Q2 | 0.92 (0.76-1.12) |  | 0.92(0.75-1.13) |  |
|  | Q3 | 0.73 (0.60-0.87) |  | 0.70 (0.57-0.85) |  |
|  | Q4 | 0.66 (0.55-0.78) |  | 0.61 (0.50-0.73) |  |
|  | Q5 (most) | 0.61 (0.52-0.72) |  | 0.52 (0.43-0.62) |  |
| Location type | Home (ref) | 1.00 | <.001 | 1.00 | <.001 |
|  | Public | 2.58 (2.23-2.98) |  | 2.28 (1.94-2.67) |  |
|  | Other (Including Aged Care and Healthcare Facilities) | 2.14 (1.62-2.82) |  | 2.41 (1.80-3.23) |  |
| Bystander Witnessed | No (ref) | 1.00 | <.001 | 1.00 | <.001 |
|  | Yes | 1.84 (1.67-2.02) |  | 1.69 (1.52-1.87) |  |

^#^adjusted for sex, age, ethnicity, rurality, deprivation, event location type and witnessed status using Firth-penalised logistic regression. Ref – reference variable; uOR – unadjusted odds ratio; aOR – adjusted odds ratio; 95%CI – 95% confidence interval; Q1-Q5 – Quintile 1 to Quintile 5.

##### Supplementary Table 3 - Associations with Bystander CPR in cases with first arresting shockable rhythms only

|  |  | Unadjusted Odds Ratio (uOR) (95%CI) | uOR P-value | Adjusted Odds Ratio^#^ (aOR) (95%CI) | aOR P-value |
| --- | --- | --- | --- | --- | --- |
| Sex | Female | 0.95 (0.77-1.17) | .600 | 1.11 (0.89-1.39) | .360 |
|  | Male (ref) | 1.00 |  | 1.00 |  |
| Age | (Years) | 0.98 (0.98-0.99) | <.001 | 0.98 (0.98-0.99) | <.001 |
| Ethnicity | European and Other (ref) | 1.00 | .976 | 1.00 | .690 |
|  | Māori | 1.01 (0.81-1.26) |  | 1.11 (0.87-1.43) |  |
|  | Pacific Peoples | 0.97 (0.72-1.32) |  | 1.08 (0.77-1.52) |  |
| Rurality | Urban (ref) | 1.00 | .010 | 1.00 | -<.001 |
|  | Rural | 1.34 (1.07-1.66) |  | 1.53 (1.21-1.94) |  |
| Deprivation (Patient address) | Q1 (least) (ref) | 1.00 | <.001 | 1.00 | <.001 |
|  | Q2 | 0.84 (0.59-1.18) |  | 0.91 (0.64-1.31) |  |
|  | Q3 | 0.66 (0.47-0.91) |  | 0.67 (0.48-0.94) |  |
|  | Q4 | 0.63 (0.46-0.87) |  | 0.59 (0.42-0.82) |  |
|  | Q5 (most) | 0.52 (0.39-0.70) |  | 0.45 (0.33-0.63) |  |
| Location Type | Home (ref) | 1.00 | <.001 | 1.00 | <.001 |
|  | Public | 2.70 (2.16-3.38) |  | 2.61 (2.05-3.33) |  |
|  | Other (Including Aged Care and Healthcare Facilities) | 1.69 (1.00-2.88) |  | 2.16 (1.22-3.82) |  |
| Bystander Witnessed | No (ref) | 1.00 | <.001 | 1.00 | <.001 |
|  | Yes | 1.91 (1.59-2.30) |  | 1.73 (1.42-2.11) |  |

^#^adjusted for sex, age, ethnicity, rurality, deprivation, event location type and witnessed status using Firth-penalised logistic regression. Ref – reference variable; uOR – unadjusted odds ratio; aOR – adjusted odds ratio; 95%CI – 95% confidence interval; Q1-Q5 – Quintile 1 to Quintile 5.

##### Supplementary Table 4 - Associations between Bystander defibrillation in all first arresting rhythm cases

|  |  | Unadjusted Odds Ratio (uOR) | uOR P-value | Adjusted Odds Ratio^#^ (aOR) | aOR P-value |
| --- | --- | --- | --- | --- | --- |
| Sex | Female | 0.35 (0.26-0.47) | <.001 | 0.61 (0.44-0.84) | <.05 |
|  | Male (ref) | 1.00 |  | 1.00 |  |
| Age | (Years) | 0.98 (0.98-0.99) | <.001 | 0.99 (0.98-1.00) | <.05 |
| Ethnicity | European and Other (ref) | 1.00 | .077 | 1.00 | .45 |
|  | Māori | 0.79 (0.61-1.02) |  | 0.89 (0.65-1.21) |  |
|  | Pacific Peoples | 0.73 (0.49-1.06) |  | 0.76 (0.47-1.21) |  |
| Rurality | Urban (ref) | 1.00 | .663 | 1.00 | .600 |
|  | Rural | 1.05 (0.83-1.33) |  | 1.08 (0.82-1.41) |  |
| Deprivation (Patient address) | Q1 (least) (ref) | 1.00 | <.001 | 1.00 | <.05 |
|  | Q2 | 0.67 (0.49-0.93) |  | 0.67 (0.47-0.97) |  |
|  | Q3 | 0.53 (0.38-0.74) |  | 0.58 (0.40-0.84) |  |
|  | Q4 | 0.58 (0.42-0.78) |  | 0.71 (0.50-1.01) |  |
|  | Q5 (most) | 0.38 (0.28-0.52) |  | 0.47 (0.33-0.68) |  |
| Location type | Home (ref) | 1.00 | <.001 | 1.00 | <.001 |
|  | Public | 13.15 (10.30-16.79) |  | 10.51 (8.13-13.58) |  |
|  | Other (Including Aged Care and Healthcare Facilities) | 0.56 (0.18-1.79) |  | 0.73 (0.25-2.14) |  |
| Bystander Witnessed | No (ref) | 1.00 | <.001 | 1.00 | <.001 |
|  | Yes | 3.29 (2.54-4.25) |  | 2.47 (1.85-3.29) |  |

^#^adjusted for sex, age, ethnicity, rurality, deprivation, event location type and witnessed status using Firth-penalised logistic regression. Ref – reference variable; uOR – unadjusted odds ratio; aOR – adjusted odds ratio; 95%CI – 95% confidence interval; Q1-Q5 – Quintile 1 to Quintile 5.

##### Supplementary Table 5 - Associations between bystander defibrillation in cases with shockable first arresting rhythms only

|  |  | Unadjusted Odds Ratio (uOR) | uOR P-value | Adjusted Odds Ratio^#^ (aOR) | aOR P-value |
| --- | --- | --- | --- | --- | --- |
| Sex | Female | 0.56 (0.42-0.75) | <.001 | 0.83 (0.59-1.15) | .260 |
|  | Male (ref) | 1.00 |  | 1.00 |  |
| Age |  | 0.99 (0.98-1.00) | <.05 | 0.99 (0.99-1.00) | .110 |
| Ethnicity | European and Other (ref) | 1.00 | .116 | 1.00 | .32 |
|  | Māori | 0.80 (0.61-1.04) |  | 0.87 (0.63-1.21) |  |
|  | Pacific Peoples | 0.74 (0.50-1.10) |  | 0.70 (0.44-1.14) |  |
| Rurality | Urban (ref) | 1.00 | .536 | 1.00 | .49 |
|  | Rural | 1.08 (0.85-1.37) |  | 1.10 (0.83-1.46) |  |
| Deprivation (Patient address) | Q1 (least) (ref) | 1.00 | <.001 | 1.00 | <.05 |
|  | Q2 | 0.72 (0.52-1.01) |  | 0.75 (0.52-1.10) |  |
|  | Q3 | 0.61 (0.43-0.85) |  | 0.61 (0.42-0.89) |  |
|  | Q4 | 0.67 (0.49-0.92) |  | 0.81 (0.57-1.16) |  |
|  | Q5 (most) | 0.48 (0.35-0.65) |  | 0.54 (0.37-0.79) |  |
| Location Type | Home (ref) | 1.00 | <.001 | 1.00 | <.001 |
|  | Public | 7.82 (6.10-10.03) |  | 7.46 (5.75-9.67) |  |
|  | Other (Including Aged Care and Healthcare Facilities) | 0.64 (0.20-2.04) |  | 0.77 (0.26-2.29) |  |
| Bystander Witnessed | No (ref) | 1.00 | <.05 | 1.00 | .05 |
|  | Yes | 1.51 (1.16-1.97) |  | 1.35 (1.00-1.83) |  |

^#^adjusted for sex, age, ethnicity, rurality, deprivation, event location type and witnessed status using Firth-penalised logistic regression. Ref – reference variable; uOR – unadjusted odds ratio; aOR – adjusted odds ratio; 95%CI – 95% confidence interval; Q1-Q5 – Quintile 1 to Quintile 5.
